# Neuropathy in a Patient With Congenital Absence of the Inferior Vena Cava

**DOI:** 10.7759/cureus.58321

**Published:** 2024-04-15

**Authors:** Karen Inzirillo, Olivia Mattner, Octavio Carranza, Marc A Swerdloff

**Affiliations:** 1 Neurology, Baptist Health South Florida, Boca Raton, USA; 2 Neurology, Florida Atlantic University Charles E. Schmidt College of Medicine, Boca Raton, USA; 3 Neurology, Boca Raton Regional Hospital, Marcus Neuroscience Institute, Boca Raton, USA

**Keywords:** congenital absence of the inferior vena cava, paresthesia, chronic venous insufficiency (cvi), neuropathy, deep vein thrombosis (dvt)

## Abstract

Neuropathic pain is a frequent complaint in the neurology clinic. We present a case of a 31-year-old male with congenital absence of the inferior vena cava (AIVC) resulting in venous hypertension who complained of lower extremity pain interfering with his daily activities. His AIVC was thought to be incidental rather than causative of his pain complaints. His examination was consistent with peripheral neuropathy. Simple lifestyle adaptations, such as restriction of physical activity and leg elevation, were sufficient to relieve his symptoms. Recognition of the role of AIVC may have prevented additional invasive procedures in our patient.

## Introduction

The absence of the inferior vena cava (AIVC) is an uncommon anomaly. Other names are IVC atresia, agenesis, or aplasia. It can be congenital or acquired. It is seen in one percent of the population, usually diagnosed in the third and fourth decades [1,2]. The most common presentation is bilateral leg pain and swelling due to deep vein thrombosis (DVT) [1,2,3]. We report a case of a 31-year-old male with a known diagnosis of congenital AIVC with bilateral leg pain and intermittent paresthesia of both feet that was exacerbated with physical activity. We attribute his neuropathy to limited venous capacity and resultant chronic venous hypertension arising from AIVC.

## Case presentation

**Figure 1:**
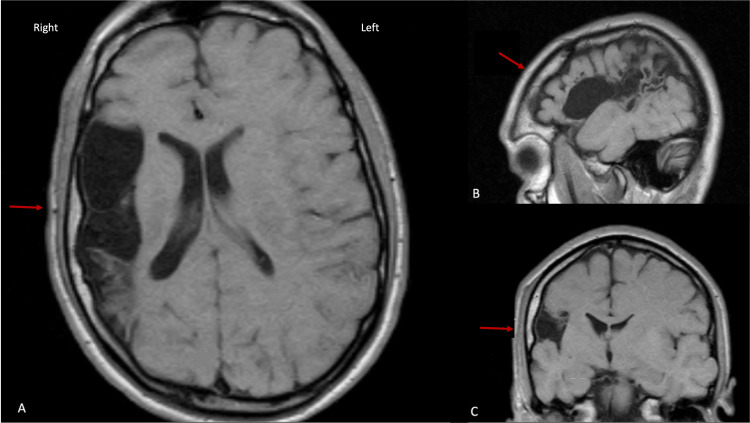
MRI brain (T1 weighted). Panel A: Axial. Smaller right hemisphere compared to the left secondary to intrauterine stroke on axial view. There is ipsilateral thickening of the skull (arrow) resulting in a Dyke-Davidoff-Masson syndrome. Panel B: Parasagittal view. Panel C: Coronal view.

A 31-year-old male presented to our office with bilateral leg paresthesia characterized as transient tingling during exertion. He had no motor deficit. He had asymptomatic hemiatrophy (Dyke-Davidoff-Mason syndrome; see Figure 1) from an intrauterine stroke. At 14 years of age, he first experienced uncomfortable tingling in his lower extremities when playing soccer. There were no sensations of burning, shooting, squeezing, or pressure. He had known AIVC, but his referring physicians thought that this was incidental to his complaints. He knew that if he restricted his physical activity, he could avoid leg pain. At age 20, he had acute bilateral leg swelling with the inability to walk secondary to bilateral deep vein thrombosis in his legs. His job required him to walk distances wherein he developed bilateral thigh and calf pain with tingling in his feet. He found that his symptoms were relieved by sitting or elevating his legs. He had stocking hypoalgesia on examination with hypoactive knee and ankle reflexes. He had normal distal and proximal leg strength (5/5 on the Medical Research Council Scale). There were no signs of leg edema.

**Figure 2:**
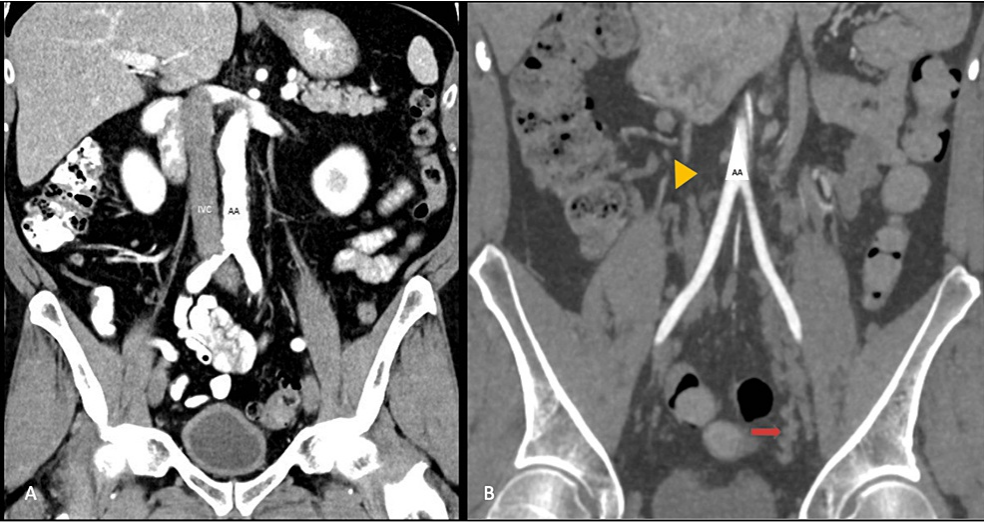
Panel A: CT scan of a normal anatomy with the abdominal aorta (AA) anterior to the inferior vena cava (IVC). Panel B: CT scan with absent IVC (yellow arrowhead) and extensive collateral venous system in the lateral pelvis (red arrow).

An ultrasound a year prior revealed a partial thrombus of the bilateral common femoral veins, right distal femoral vein, and left popliteal vein. Nerve conduction studies were normal. CT scan of the abdomen and pelvis demonstrated AIVC with extensive collateral veins below the level of the kidneys (Figure 2). A thrombophilia workup was normal. Multiple nerve blocks in the spine for lumbar radiculopathy and a subsequent lumbar laminectomy failed to relieve his symptoms. Our recommendations were to restrict his walking and encourage leg elevation to enhance venous return. We felt that intermittent over-exertion resulted in a transient increase in venous hypertension precipitating uncomfortable paresthesia.

## Discussion

The venous system of the lower extremities is composed of deep, superficial, and perforator veins. Normally, the venous flow is propelled by muscle contraction forcing blood into veins with valves that facilitate a one-way return of blood to the heart via the IVC. Venous stasis may be caused by venous obstruction, external venous compression, incompetent valves with reflux, and maldevelopment of venous structures [4] as in congenital AIVC. 

Chronic venous insufficiency (CVI) is seen in the elderly and obese with a prevalence of up to 40% [4,5]. The main cause is venous reflux and/or obstruction causing leg swelling and pain. Other symptoms reported are leg heaviness (70%), pain (50%), pruritis, or paresthesia (20%) [5]. Leg pain usually increases with the progression of the disease and negatively affects the quality of life [5]. Leg pain may result from inflammatory mediators, such as adhesion molecules, cytokines, pro-thrombotic agents, and prostaglandins. It has been proposed that neuropathy and neuropathic pain in CVI may result from venous microangiopathy from increased endoneurial pressure [5]. CVI may lead to deep vein thrombosis (DVT), pulmonary embolism (PE), post-thrombotic syndrome (PTS), skin hyperpigmentation, lipodermatosclerosis, and ulceration [5]. 

In congenital AIVC, venous hypertension results from the absence of the largest venous pathway to the heart, the IVC, (yellow arrowhead in Figure 2) resulting in the enlargement of distal smaller collateral venous channels (red arrow in Figure 2). These collateral veins have competent valves. Venous stasis occurs when strenuous exercise increases cardiac output overwhelming the capacity of the smaller caliber collateral system.

Descriptions of leg pain in AIVC are vague. One study listed 12 of 17 cases with “pain” [3]. Pain from peripheral neuropathy is described as shooting, squeezing, burning, or tingling. Painless sensory ataxia may be present with or without feeling numbness. In our case, neuropathy occurred in the second decade of life. He was symptomatic after venous capacity was exceeded in the absence of sufficiently large venous channels. We recommended leg elevation for 30 minutes every three hours of work as noted in the literature [5]. He will require lifelong anticoagulation to prevent DVT formation [2]. Compression stockings will not be useful since they will not overcome inadequate venous anatomy or reduce PTS [6]. Fatal pulmonary embolism is of low risk since small caliber collateral veins filter out large emboli bound for the lungs [2].

## Conclusions

In congenital AIVC, venous hypertension presents at birth without the comorbidities seen in late-onset CVI. Our patient was an avid young athlete, which caused earlier symptom onset than other reported cases of AIVC. Frequent demands on his venous system caused increased endoneurial pressure and earlier development of venous microangiopathy from venous stasis. This is the hypothesized mechanism of neuropathic pain in CVI. We propose that congenital AIVC is responsible for our patient's peripheral neuropathy. Transient paresthesia was experienced when increased cardiac output during exertion exceeds the return capacity of an inadequate venous collateral system.

Pain in acquired venous insufficiency has been inadequately described. Peripheral neuropathy in the setting of congenital AIVC is underrecognized and may impact the quality of life. In this situation, we recommend lifestyle changes that limit symptoms to an acceptable level and to avoid invasive and ultimately ineffective procedures.
